# Selective serotonin reuptake inhibition modulates response inhibition in Parkinson’s disease

**DOI:** 10.1093/brain/awu032

**Published:** 2014-02-27

**Authors:** Zheng Ye, Ellemarije Altena, Cristina Nombela, Charlotte R. Housden, Helen Maxwell, Timothy Rittman, Chelan Huddleston, Charlotte L. Rae, Ralf Regenthal, Barbara J. Sahakian, Roger A. Barker, Trevor W. Robbins, James B. Rowe

**Affiliations:** 1 Department of Clinical Neurosciences, University of Cambridge, Cambridge, UK; 2 Department of Experimental Psychology, University of Cambridge, Cambridge, UK; 3 Medical Research Council Cognition and Brain Sciences Unit, Cambridge, UK; 4 Division of Clinical Pharmacology, Rudolf-Boehm-Institute of Pharmacology and Toxicology, University of Leipzig, Leipzig, Germany; 5 Behavioural and Clinical Neuroscience Institute, Cambridge, UK

**Keywords:** Parkinson’s disease, response inhibition, serotonin, citalopram, functional MRI

## Abstract

Impulsivity is common in Parkinson’s disease even in the absence of impulse control disorders. It is likely to be multifactorial, including a dopaminergic ‘overdose’ and structural changes in the frontostriatal circuits for motor control. In addition, we proposed that changes in serotonergic projections to the forebrain also contribute to response inhibition in Parkinson’s disease, based on preclinical animal and human studies. We therefore examined whether the selective serotonin reuptake inhibitor citalopram improves response inhibition, in terms of both behaviour and the efficiency of underlying neural mechanisms. This multimodal magnetic resonance imaging study used a double-blind randomized placebo-controlled crossover design with an integrated Stop-Signal and NoGo paradigm. Twenty-one patients with idiopathic Parkinson’s disease (46–76 years old, 11 male, Hoehn and Yahr stage 1.5–3) received 30 mg citalopram or placebo in addition to their usual dopaminergic medication in two separate sessions. Twenty matched healthy control subjects (54–74 years old, 12 male) were tested without medication. The effects of disease and drug on behavioural performance and regional brain activity were analysed using general linear models. In addition, anatomical connectivity was examined using diffusion tensor imaging and tract-based spatial statistics. We confirmed that Parkinson’s disease caused impairment in response inhibition, with longer Stop-Signal Reaction Time and more NoGo errors under placebo compared with controls, without affecting Go reaction times. This was associated with less stop-specific activation in the right inferior frontal cortex, but no significant difference in NoGo-related activation. Although there was no beneficial main effect of citalopram, it reduced Stop-Signal Reaction Time and NoGo errors, and enhanced inferior frontal activation, in patients with relatively more severe disease (higher Unified Parkinson’s Disease Rating Scale motor score). The behavioural effect correlated with the citalopram-induced enhancement of prefrontal activation and the strength of preserved structural connectivity between the frontal and striatal regions. In conclusion, the behavioural effect of citalopram on response inhibition depends on individual differences in prefrontal cortical activation and frontostriatal connectivity. The correlation between disease severity and the effect of citalopram on response inhibition may be due to the progressive loss of forebrain serotonergic projections. These results contribute to a broader understanding of the critical roles of serotonin in regulating cognitive and behavioural control, as well as new strategies for patient stratification in clinical trials of serotonergic treatments in Parkinson’s disease.

## Abstract (precis)

Impulsivity is common in Parkinson’s disease. In a double-blind, placebo-controlled study with multi-modal imaging, Ye *et al*. reveal improved response inhibition in some patients receiving the SSRI citalopram, including those with advanced disease. Improvements correlated with preserved frontostriatal structural connectivity and drug-induced prefrontal activity, highlighting the need for patient stratification in trials.

## Introduction

The problem of impulsivity in Parkinson’s disease is highlighted by the severity of impulse control disorders in 13.6% of patients (Weintraub *et al.*, 2010). Impulsive actions (e.g. unsuccessful response inhibition, premature response) and choices (e.g. reward/risk-based decisions) exist even in the absence of impulse control disorders (Voon *et al.*, 2011*a*; Bentivoqlio *et al.*, 2012; Nombela *et al.*, 2014) and are likely to be multifactorial. This study focuses on the serotonergic mechanisms of response inhibition, rather than the dopaminergic mechanisms of choice impulsivity.

In addition to dopaminergic ‘overdose’ (Rowe *et al.*, 2008; Voon *et al.*, 2011*b*) and structural changes in the frontostriatal circuits for motor control and decision-making (Rae *et al.*, 2012), we propose that changes in serotonergic projections to the forebrain also exacerbate the impairment of response inhibition (Politis *et al.*, 2010, 2012). Enhancing central serotonin transmission with selective serotonin reuptake inhibitors (SSRIs) might therefore provide an adjunctive treatment for motor impulsivity in Parkinson’s disease. Citalopram is a SSRI that increases the extracellular levels of serotonin in the prefrontal cortex ∼4-fold, but not levels of noradrenaline or dopamine (Bymaster *et al.*, 2002). We therefore investigated whether citalopram can improve response inhibition and enhance its neural systems in Parkinson’s disease.

We studied two forms of response inhibition: NoGo and Stop-Signal inhibition. NoGo inhibition requires action selection and restraint mechanisms for the prevention of a prepotent response, whereas Stop-Signal tasks invoke the cancellation of an initiated response and induce neuronal inhibition of motor actions. There is strong preclinical evidence from animal and human studies that serotonin plays an important role in regulating action restraint. For example, in rats and humans, enhancing cortical serotonin transmission (e.g. using the SSRI citalopram or serotonin receptor agonists) reduces impulsive responses (Homberg *et al.*, 2007) and increases NoGo activations in the right inferior frontal gyrus (Anderson *et al.*, 2002; Del-Ben *et al.*, 2005; Völlm *et al.*, 2006; Macoveanu *et al.*, 2013). Serotonin efflux is also enhanced naturally in the dorsal raphe nucleus, which projects to the forebrain, when rats perform a task that requires waiting and action restraint (Miyazaki *et al.*, 2011). In contrast, depleting central serotonin (e.g. by acute tryptophan depletion or a serotonin receptor antagonist M100907) increases premature responding (Fletcher *et al.*, 2007; Robinson *et al.*, 2008), impairs NoGo inhibition and right inferior frontal gyrus activation (Harrison *et al.*, 1999; Rubia *et al.*, 2005). In addition, several clinical disorders are associated with both impulsivity and reduction or deregulation of serotonin transmission, including Parkinson’s disease, attention deficit hyperactivity disorder (Zepf *et al.*, 2008), frontotemporal dementia (Huey *et al.*, 2006), Tourette’s syndrome (Haugbøl *et al.*, 2007) and obsessive-compulsive disorder (Chamberlain *et al.*, 2005).

In contrast, the link between serotonin and action cancellation is less well established. Animal and human studies have often not shown an effect of serotonin on Stop-Signal tasks (as measured by the Stop-Signal reaction time, SSRT) (Clark *et al.*, 2005; Chamberlain *et al.*, 2006, 2007*b*; Bari *et al.*, 2009; Eagle *et al.*, 2009; Drueke *et al.*, 2010; Nandam *et al.*, 2011). However, the impact of serotonin manipulations on behaviour and cortical activation depends on individual differences in the serotoninergic system and susceptibility to impulsive behaviour (Crean *et al.*, 2002; Macoveanu *et al.*, 2013). In the later stages of Parkinson’s disease, therefore, the depletion of serotonin and other monoamine neurotransmitters to the frontal cortex and striatum (Politis *et al.*, 2010, 2012; Goldstein *et al.*, 2011) may predispose to a therapeutic effect of serotonin reuptake inhibition on action cancellation.

In this pharmacological functional MRI study, we examined the effect of citalopram (30 mg versus placebo) on response inhibition in Parkinson’s disease. The task incorporated NoGo and Stop-Signal trials while patients took their usual dopaminergic medication. We predicted that (i) both NoGo and Stop-Signal inhibition is impaired in Parkinson’s disease (*cf*. Gauggel *et al.*, 2004; Obeso *et al.*, 2011; Nombela *et al.*, 2014); (ii) the behavioural effect of citalopram would depend on patients’ disease severity (*cf.* Cilia *et al.*, 2011); and (iii) the behavioural effect may also relate to enhancement of frontal cortical activation after citalopram. We additionally examined other potential predictors of patients’ responsiveness to citalopram, such as preserved structural connections within the frontostriatal networks, by using diffusion tensor imaging.

## Materials and methods

### Participants

Twenty-one patients with idiopathic Parkinson’s disease (UK Parkinson’s Disease Society Brain Bank Clinical Diagnostic Criteria) were recruited through the Cambridge University Parkinson’s Disease Research Clinic. Inclusion criteria were: (i) Hoehn and Yahr stage 1.5–3; (ii) age 45–80 years; (iii) English speaking; (iv) right-handed; and (v) non-demented at last assessment (clinical impression and Mini-Mental State Examination score >26/30). Patients were excluded by (i) clinically significant current depression; (ii) contraindications to MRI or citalopram; (iii) epilepsy or past significant psychiatric disorders. None of our patients met criteria for an impulse control disorder. All were tested on regular dopaminergic medication, including levodopa (*n = *19), non-ergot dopamine agonists (*n = *17: eight pramipexole, eight ropinirole, one rotigotine), and other anti-parkinsonian medication (*n = *8: seven amantadine, one rasagiline, one selegiline). Levodopa equivalent dose was calculated by the formula of Tomlinson *et al.* (2010). Demographic and clinical features of participants are given in Table 1.

**Table 1:** Demographic and clinical features, and neuropsychological measures (means, standard deviations and group differences)

Features / Measures	Parkinson’s disease	Control	Group difference
Male:female	11:10	12:8	n.s.
Age, years	64.0 (8.1)	65.3 (5.7)	n.s.
Education, years	14.6 (3.8)	15.1 (2.5)	n.s.
Mini-Mental State Examination	28.9 (1.2)	29.3 (0.9)	n.s.
Duration of disease, years	10.8 (4.9)	–	–
UPDRS (ON medication)			
I: mentation, behaviour and mood	8.8 (4.9)	–	–
III: motor	20.6 (7.7)	–	–
Hoehn and Yahr	1.9 (0.4)	–	–
Schwab and England Activities of Daily Living Scale	86.5 (5.9)	–	–
Levodopa actual dose, mg/day	393.4 (221.0)		
Levodopa equivalent dose, mg/day	632.6 (310.6)	–	–
Beck Depression Inventory II	9.9 (5.5)	3.8 (3.9)	<0.001
REM Sleep Behaviour Disorder	6.4 (2.9)	2.0 (1.5)	<0.001
Category fluency test	20.6 (4.9)	24.3 (6.7)	0.05
Letter fluency test	17.0 (5.6)	18.1 (4.5)	n.s.
Forward digit span	7.0 (1.1)	7.2 (0.8)	n.s.
Backward digit span	5.4 (1.5)	5.7 (1.3)	n.s.
Simple reaction time task			
Correct trials, %	98.9 (1.2)	99.0 (1.3)	n.s.
Reaction time, ms	293.7 (53.0)	314.4 (71.8)	n.s.
Choice reaction time			
Correct trials, %	98.9 (1.3)	99.7 (0.5)	<0.05
Reaction time, ms	353.4 (47.3)	392.2 (70.0)	<0.05

Group difference: *P*-values of chi-squared or two-sample two-tailed *t*-tests, as appropriate; n.s. = not significant, *P* > 0.1.

Twenty healthy control subjects with no history of significant neurological or psychiatric disorders were recruited from the Parkinson’s Disease Research Clinic database and the volunteer panel of the MRC Cognition and Brain Sciences Unit. This study was approved by the local research ethics committee and exempted from Clinical Trials status by the Medicines and Healthcare products Regulatory Authority. Written informed consent was obtained from all participants.

### Experimental design

**Figure 1:**
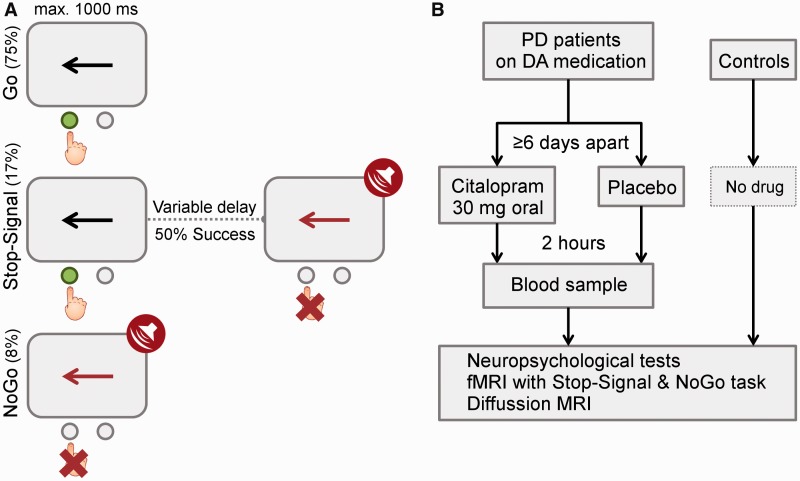
(**A**) Task and stimuli. (**B**) Design and drug. PD = Parkinson’s disease; DA = dopamine.

This study used a double-blind randomized crossover design (Figure 1). Separate sessions at least 6 days apart included a neuropsychological battery and brain imaging, after either a 30 mg oral citalopram or an identically over-coated placebo capsule. Nineteen patients completed both sessions. Blood samples were taken 2 h after administration, immediately before functional MRI scanning to coincide with the estimated time of peak plasma concentration (Sangkuhl *et al.*, 2011). Mean plasma concentration under citalopram: 46 ng/ml, range 11–70 mg/ml; under placebo: 0 mg/ml. Control subjects had one testing session only without drug.

The task included randomly interleaved NoGo and Stop-Signal trials (figure 1), which enabled us to examine both types of inhibition while matching for drug levels, practice effects and fatigue. There were 360 Go trials (75%), 40 NoGo trials (8%) and 80 Stop-Signal trials (17%). On Go trials, subjects responded to a left/right black arrow (1000 ms) by pressing buttons with their right hand. Responses were made with either index finger (for the left arrow) or middle finger (for the right arrow). In Stop-Signal trials, a response was initially cued by the left/right black arrow but the arrow colour changed to red concurrent with a tone after a Stop-Signal delay and subjects were required to not make the response. The Stop-Signal delay was varied from trial to trial by using a step-up/down algorithm with an initial estimate of 250 ms to maintain 50% successful inhibition (Chamberlain *et al.*, 2007*a*). This is similar to the tracking algorithm used in previous imaging studies of the Stop-Signal task (Cubillo *et al.*, 2012; Pauls *et al.*, 2012; Costa *et al.*, 2013). In NoGo trials, subjects were required to make no response to the red arrow (1000 ms) and beep, equivalent to a Stop-Signal delay of zero.

Subjects also completed the Beck Depression Inventory II, REM Sleep Behaviour Disorder, category (‘animals’, ‘items of clothing’ or ‘fruit/vegetables’) and letter fluency (‘P’, ‘S’ or ‘F’), digit span, and manual simple (one stimulus type, one response button) and choice reaction time tasks (two stimulus types, two response buttons; see Supplementary material for a detailed description). The versions of each fluency task were randomly given across drug and placebo sessions of each subject, after pilot studies demonstrated their equivalence in older adults.

We measured four behavioural parameters of interest in the functional MRI task: (i) mean reaction time of correct Go trials; (ii) SSRT; (iii) the rate of Go commission errors; and (iv) the rate of NoGo commission errors. A NoGo commission error means that participants pressed a button. A Go commission error means that participants pressed a wrong button. The SSRT was estimated using the integration method (Logan and Cowan, 1984), and adjusted for omission errors on Go trials using Equation 1 below, where *P*(respond | signal) is the probability of responding. The Go omission rate was 1% in controls, 7% in Parkinson’s disease-placebo, and 6% in Parkinson’s disease-citalopram (Parkinson’s disease-placebo versus control: two-sample *t*-test, *P < *0.01; Parkinson’s disease-citalopram versus Parkinson’s disease-placebo: paired-sample *t*-test, not significant). The distribution of Go reaction times indicated that the omission errors were not a result of exceeding the response window, but rather motor impairments (e.g. slipping off the buttons or failure to press firmly). The omission rate of individual patients was used to adjust SSRT because the omission errors may lead to an underestimation of SSRT.
(1)
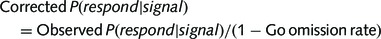



Disease effects were tested with two-sample *t*-tests, comparing Parkinson’s disease-placebo with controls. In this study, the ‘disease effect’ refers to treated Parkinson’s disease (*cf.* Lewis *et al.*, 2003; Weintraub *et al.*, 2011). In principle, differences between Parkinson’s disease-placebo and control subjects could be because of the presence of Parkinson’s disease, the use of anti-parkinsonian medication, or the additional use of a placebo tablet in the patient group. However, the concurrent use of dopaminergic drugs is unlikely to fully explain the group differences (Obeso *et al.*, 2011).

Analysis of drug effects used repeat-measures ANOVAs with drug (citalopram versus placebo) as a within-subject factor and disease severity [Unified Parkinson’s Disease Rating Scale (UPDRS) motor score], levodopa equivalent dose, age and plasma concentration of citalopram as covariates.

### Functional magnetic resonance imaging data acquisition and analysis

Functional images were acquired using a quiet echo planar imaging (EPI) sequence with a Siemens Trio 3 T scanner (2656-ms repetition time, 44-ms echo time, 78° flip angle, 32 sequential descending oblique axial slices, 192 × 192 mm^2^ field of view, 3-mm thickness, 0.75-mm slice gap, and 3 × 3 mm^2^ in-plane resolution; (Peelle *et al.*, 2010). High-resolution T_1_-weighted MPRAGE images were also acquired (144 sequential sagittal slices, 240 × 240 mm^2^ field of view, 1.25-mm thickness, and 1.25 × 1.25 mm^2^ in-plane resolution).

Functional MRI preprocessing and analysis used SPM8 (www.fil.ion.ucl.ac.uk/spm). Eleven volumes were discarded to allow magnetization equilibration. Functional images were realigned to the first image, sinc-interpolated to correct for differences in slice acquisition time, normalized to the Montreal Neurological Institute (MNI) template using iterative segmentation, and denoised with a wavelet-based approach (Khullar *et al.*, 2011).

General linear models were estimated for each subject by convolving a design matrix with the canonical haemodynamic response function. The design matrix included discrete events for correct Stop-Signal, NoGo and Go trials, commission errors (Stop-Signal, NoGo and Go trials) and omission errors (Go trials). Six standard movement parameters (*x*, *y*, *z*, pitch, roll, and yaw) were included as nuisance regressors. Classical parameter estimation was applied with a lag−1 autoregressive model and high-pass filter of 128 s. Contrast images of interest were computed for each subject: Stop-Signal, NoGo and Go versus implicit baseline, Stop-Signal > Go and NoGo > Go. The contrasts were entered into second-level ‘random-effect’ models to examine the effects of disease and citalopram on event-related activations.

Activation maps of the Stop-Signal > Go and NoGo > Go contrasts were computed for each group (one-sample *t*-tests, voxel-level *P < *0.001 uncorrected, and cluster-level *P < *0.05 family-wise-error correction for multiple comparison, FWE). We also constructed a study-specific region of interest for the right inferior frontal gyrus, in view of our previous hypotheses of the task-related activation and effects of Parkinson’s disease. This region included voxels from the Automated Anatomical Labelling (AAL)-defined right inferior frontal gyrus that were activated by Stop-Signal>Go or NoGo>Go contrasts in control subjects. This mask was used for small volume correction for multiple comparisons (voxel-wise *P < *0.05 FWE-corrected).

The right inferior frontal gyrus mask was also used for a region of interest analysis in which parameter estimates (betas from subject-level models) were pooled (i) for repeated-measures ANOVAs with drug as a within-subject factor and UPDRS, levodopa equivalent dose, age and drug concentration as covariates; and (ii) for correlations between changes in behaviour and changes in right inferior frontal gyrus activation.

### Diffusion magnetic resonance imaging

We additionally examined white matter connections of the frontostriatal circuits as a potential predictor of the citalopram effect on behavioural performance. Diffusion-weighted images were collected along 63 gradient directions (single acquisition, 63 sequential ascending axial slices, 192 × 192 mm^2^ field of view, 2-mm thickness, and 2 × 2 mm^2^ in-plane resolution) and analysed with FSL4.1 (www.fmrib.ox.ac.uk/fsl). Images were corrected for head movements and eddy currents, and smoothed with a 2.5-mm Gaussian kernel. Diffusion tensors were linearly fitted to the diffusion-weighted imaging, and fractional anisotropy maps were calculated for tract-based spatial statistics (Smith *et al.*, 2006). Fractional anisotropy images were corrected for outlier values, registered to the FMRIB58 fractional anisotropy standard-space image, and normalized to MNI 152 space. A mean fractional anisotropy skeleton was derived and thresholded at fractional anisotropy > 0.2 to represent the centre of the white matter tracts common to all subjects. Permutation tests with threshold-free cluster enhancement (*P < *0.05 TFCE-corrected) (Smith and Nichols, 2009) examined whether citalopram’s effects on behavioural performance (reduction in SSRT or NoGo error) varied with frontostriatal connectivity.

## Results

### Behavioural results

Details of subject demographics, clinical disease severity and neuropsychological scores are given in Table 1. The groups were well matched for age, sex and educational years. Higher scores on the Beck Depression Inventory-II and REM Sleep Behaviour Disorder questionnaires are typical of mild to moderate Parkinson’s disease. Note that higher scores on the Beck Depression Inventory-II do not in themselves establish a diagnosis of depression. The groups were also matched in reaction time and accuracy of the simple reaction time task. In the choice reaction time task, the patient group was faster but significantly less accurate than the control group (*cf*. Hughes *et al.*, 2010).

Table 2 shows the SSRT, Go reaction time, Go error rates, and NoGo error rates from control subjects and from patients under citalopram and placebo, during performance of the functional MRI paradigm. Compared with control subjects, the Parkinson’s disease-placebo group had longer SSRTs, more NoGo errors and Go errors. The group difference in SSRT could not be attributed to strategic slowing of responses, given the similarity of Go reaction time.

**Table 2:** Performance on the Stop-Signal and NoGo task

Parameters	Control	Parkinson’s disease		Disease effects	Drug effects	
		Placebo	Citalopram		Drug	Drug × UPDRS
SSRT (ms)	142 (44)	167 (50)	180 (75)	*P < *0.05	*P < *0.05	*P < *0.01
Go reaction time (ms)	532 (129)	554 (108)	555 (100)	n.s.	n.s.	n.s.
NoGo error	0.01 (0.01)	0.04 (0.04)	0.05 (0.09)	*P < *0.01	n.s.	*P < *0.05
Go error	0.01 (0.01)	0.02 (0.02)	0.02 (0.02)	*P < *0.001	n.s.	n.s.

Values are group means (standard deviations). Disease effects refer to the contrast ‘Parkinson’s disease-placebo versus control’ of two-sample *t*-tests (one-tailed). Drug effects refer to the main effect of drug (Parkinson’s disease-citalopram versus Parkinson’s disease-placebo) and the interaction of drug and UPDRS (Drug × UPDRS) from repeated-measures ANOVAs (with UPDRS, age, levodopa equivalent dose and plasma concentration as covariates). n.s. = not significant, *P* > 0.1.

**Figure 2:**
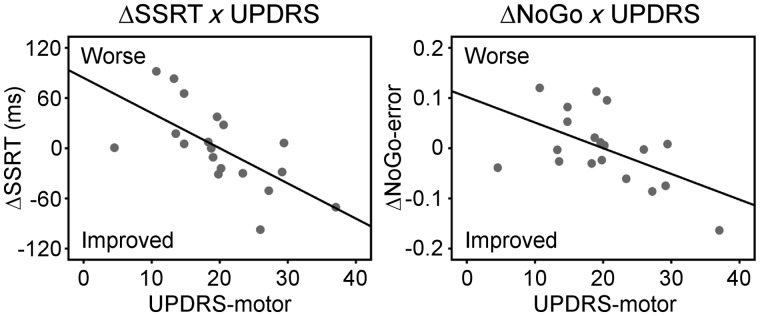
In behaviour, citalopram reduced SSRT (*left*) and NoGo error rate (*right*) in relatively more advanced disease. The drug-induced changes of SSRT (ΔSSRT) and NoGo-error (ΔNoGo-error) negatively correlated with UPDRS-motor score (*P < *0.05).

Figure 2 shows the behavioural effects of citalopram on response inhibition. ANOVAs revealed a significant interaction between drug and UPDRS (motor) for both SSRT [*F*(1,14) = 10.54, *P < *0.01] and NoGo error rate [*F*(1,14) = 5.71, *P < *0.05]. This indicates that citalopram reduced SSRT and NoGo error rate in relatively more advanced disease (larger UPDRS-motor score). For NoGo error rate, there was also an interaction between drug and levodopa equivalent dose [*F*(1,14) = 11.34, *P < *0.01], suggesting that ΔNoGo-error positively correlated with levodopa equivalent dose. Note that there was no correlation between patients’ UPDRS-motor score and levodopa equivalent dose (*P* > 0.9). We also included the Beck Depression Inventory-II score as a covariate in a separate ANOVA but observed no interaction between depression and drug effects.

### Functional and diffusion magnetic resonance imaging

**Figure 3:**
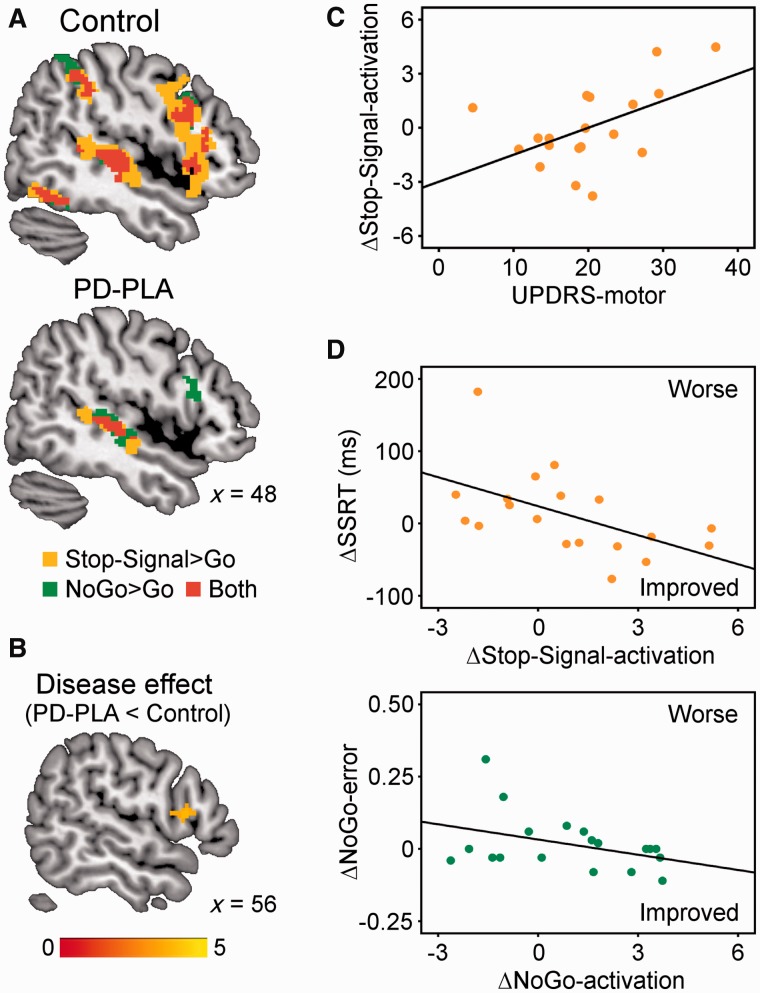
(**A**) In functional MRI, controls showed greater stop-related (Stop-Signal > Go, orange) and NoGo-related activations (NoGo > Go, green) in the right inferior frontal gyrus (*P < *0.05 corrected). Activation overlap is in red. Coordinates are in MNI space. (**B**) The stop-related right inferior frontal gyrus activation was significantly weaker in Parkinson’s disease-placebo (PD-PLA) than in controls (disease effect, *P < *0.05 corrected). Colour scale indicates *t*-values. (**C**) Citalopram’s enhancement of right inferior frontal gyrus activation in successful stops positively correlated with UPDRS-motor score (*P < *0.05). (**D**) The drug-induced changes of SSRT (ΔSSRT) and NoGo-error (ΔNoGo-error) respectively related to right inferior frontal gyrus activation changes in successful Stop-Signal trials (ΔStop-Signal-activation) and those in NoGo trials (ΔNoGo-activation).

Figure 3A and B present inhibition-related activations in controls and Parkinson’s disease-placebo, and group differences. Controls showed typical activations of right inferior frontal gyrus for Stop-Signal > Go [peak coordinates in MNI space: (54 18 8), *t* = 7.17, 1621 voxels] and for NoGo > Go [peak: (48 18 −2), *t* = 6.00, 452 voxels]. The stop-related activations extended dorsally into the caudal part of the middle frontal gyrus, and adjacent premotor cortex, in keeping with previous imaging studies of Stop-Signal tasks (Rae *et al.*, 2014). Stop- and NoGo-related activations were also seen in the superior temporal cortex and ventral occipitotemporal cortex, consistent with the presence of audiovisual cues. The stop-related right inferior frontal gyrus activation was significantly reduced in Parkinson’s disease-placebo compared with controls (‘disease effect’). However, no group difference was observed on right inferior frontal gyrus activation during successful NoGo trials.

As in the behavioural data, the regional analysis on right inferior frontal gyrus activation showed an interaction between drug and UPDRS for Stop-Signal trials [*F*(1,14) = 5.07, *P < *0.05], suggesting that citalopram positively modulated right inferior frontal gyrus activation in more advanced disease (Fig. 3C). There was no main effect of drug for Stop-Signal trials (*P = *0.25) or for NoGo trials (*F* < 1).

The right inferior frontal gyrus is the focus of this study, but the Stop-Signal and NoGo tasks were also associated with activation of the supplementary motor area in our study and previous reports. We additionally analysed the drug effects on the supplementary motor area and presented as the Supplementary material. In brief, we observed no drug effect on the supplementary motor area.

To determine whether citalopram’s effect on behavioural performance can be predicted by its enhancement of right inferior frontal gyrus activation in Parkinson’s disease, we conducted a correlation with the change of SSRT (ΔSSRT: SSRT-citalopram − SSRT-placebo) and the change of right inferior frontal gyrus activation on successful Stop-Signal trials (ΔStop-Signal-activation), as well as a correlation between the change of NoGo error rate (ΔNoGo-error) and the change of right inferior frontal gyrus activation on correct NoGo trials (ΔNoGo-activation). Figure 3D shows that citalopram’s effect on both restraint (ΔNoGo-error: r = −0.39, *P = *0.05) and cancellation (ΔSSRT: r = −0.54, *P < *0.01) depended on the change in right inferior frontal gyrus activation. This effect was preserved after exclusion of a single subject with high values of ΔNoGo and ΔSSRT.

**Figure 4:**
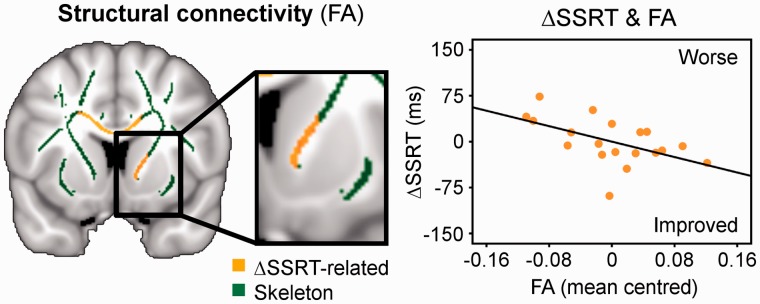
Citalopram’s effect on response inhibition was also related to the strength of structural connectivity (fractional anisotropy, FA) between the frontal cortex and striatum, showing a negative correlation of the drug-induced SSRT change (ΔSSRT) with fractional anisotropy of the skeleton voxels in the right internal capsule (mean centred). Thresholded at *P < *0.05 TFCE-corrected.

Another potential predictor of citalopram’s effect on behavioural performance was the white matter connectivity, including the fibres connecting the right frontal lobe, striatum and thalamus through the anterior internal capsule (Fig. 4). There was a negative correlation of the drug-induced SSRT change with individual patients’ fractional anisotropy of white matter in the right internal capsule. Patients with greater anatomical frontostriatal connectivity showed a drug-induced reduction in SSRT. No such relation was obtained between NoGo error and fractional anisotropy.

## Discussion

In this pharmacological functional MRI study, we used the integrated NoGo and Stop-Signal task to study action restraint and cancellation in Parkinson’s disease. The Stop-Signal and NoGo inhibition is assessed by SSRT and NoGo error rate, and associated with widespread activations including the activation on the right inferior frontal gyrus (Swick *et al.*, 2011; Rae *et al.*, 2014). As in previous patient studies (Gauggel *et al.*, 2004; Obeso *et al.*, 2011; Nombela *et al.*, 2014) the behavioural impulsivity in Parkinson’s disease was evident in the absence of clinical impulse control disorders: our cohort had longer SSRT and more NoGo errors than controls. A single dose of the selective serotonin reuptake inhibitor citalopram has the potential to improve response inhibition, but only in a subset of patient with Parkinson’s disease.

Citalopram had no beneficial behavioural effect when averaged across all patients. The impact of citalopram on response inhibition (reducing SSRT and NoGo errors) was beneficial in patients with relatively more advanced disease (larger UPDRS-motor score) and related to the effect of citalopram on activation of the right inferior frontal gyrus. We cannot be certain whether the effect of serotonergic agents is direct, by selective serotonin ‘restoration’, or indirect, through serotonergic interactions with dopamine, noradrenaline or *N*-methyl-D-aspartate (NMDA) (Higgins *et al.*, 2003; Winstanley *et al.*, 2005). Nonetheless, our findings reinforce preclinical and comparative studies of serotonergic modulations of response inhibition, and suggest that patients with more severe motor signs (UPDRS scores) and relatively preserved structural connectivity in frontostriatal networks (from diffusion-weighted imaging) could be stratified to receive citalopram in further clinical trials.

In the ‘Introduction’ section we highlighted the possible psychopharmacological differences between action restraint and action cancellation, with evidence for serotonergic regulation of the former, but not latter, type of inhibition (Eagle *et al.*, 2008). These observations included studies of citalopram and other SSRIs in human (Del-Ben *et al.*, 2005; Chamberlain *et al.*, 2006; Macoveanu *et al.*, 2013) and rodent versions of Stop-Signal tasks (Bari *et al.*, 2009; Eagle *et al.*, 2009) and tasks that require action restraint during waiting (Harrison *et al.*, 1999; Miyazaki *et al.*, 2011; Baarendse and Vanderschuren, 2012). This study confirmed that citalopram can reduce NoGo errors in patients with relatively advanced disease and those for whom citalopram increases right inferior frontal gyrus activation.

However, our study also showed a clear effect of citalopram on action cancellation and associated cortical activation, which, at first glance, contrasts with the literature on Stop-Signal tasks in healthy humans and animal models. However, two factors must be considered. First, is the nature of inhibitory processes required for Stop-Signal tasks and other forms of inhibitory control. Attentional reorienting to task-relevant stimuli is a feature of both NoGo and Stop-Signal trials (Levy and Wagner, 2011) but also of ‘continue’ trials, which are associated with right inferior frontal gyrus activation in the absence of inhibition (Sharp *et al.*, 2010). A failure of attending away from the active ‘go’ response following the ‘stop’ cue may relate to the impulsivity of Parkinson’s disease. Nonetheless, inhibition of actions *per se* has been linked to right inferior frontal gyrus in health (Zhang *et al.*, 2012). Moreover, reversal learning deficits after central serotonin depletion may be considered a failure to inhibit responding to a previously learned but task-irrelevant stimulus (Clarke *et al.*, 2004, 2007; Walker *et al.*, 2009). Citalopram may benefit response inhibition by targeting more than one of these processes in Stop-Signal and NoGo trials.

Second and perhaps more importantly, one must consider the relationship between the impact of serotonin manipulation and the baseline serotonin functioning. Previous studies showed that central serotonin depletion only impaired action restraint and cancellation in adults who had a family history of alcoholism and significantly lower central serotonin concentration, but not in those with a low risk of alcoholism and no family history of alcoholism (LeMarquand *et al.*, 1999; Crean *et al.*, 2002). It is possible that individuals with serotonergic deficits are more likely to respond to the acute change of serotonin transmission. This explains why we observed the behavioural benefit of citalopram in patients with Parkinson’s disease (especially those with relatively more advanced disease) but previous studies with healthy humans did not (Chamberlain *et al.*, 2006; Drueke *et al.*, 2010; Nandam *et al.*, 2011).

The observed effect of a selective reuptake inhibitor such as citalopram is unlikely to be mediated by directly increasing the cortical extracellular level of dopamine or noradrenaline, as levels of both remain at baseline after an acute citalopram administration (Bymaster *et al.*, 2002). However, postsynaptic effects of SSRIs may not be restricted to serotonin receptor signalling pathways. Recent evidence has shown interactions between serotonin 5-HT_2A_ and dopamine D_2_ receptors through forming heteromeric complexes in prefrontal and striatal regions (Borroto-Escuela *et al.*, 2010; Albizu *et al.*, 2011), between cortical 5-HT_1A_ and NMDA receptors by downregulating microtubule/kinesin-based dendritic transport of NMDA receptors (Yuen *et al.*, 2005), and between 5-HT_3_ and noradrenaline receptors in the locus coeruleus (Ortega *et al.*, 2012). We therefore cannot rule out a contribution of such secondary mechanisms to the effect of citalopram, given the correlation between levodopa dose equivalent and drug-induced reduction of NoGo errors. Such a correlation was not observed on Stop-Signal trials, consistent with the literature showing that dopaminergic influences on SSRT performance are minor (Robbins, 2007).

One may argue that acute administration of SSRIs such as citalopram may reduce serotonergic transmission through stimulating presynaptic 5-HT_1A_ inhibitory autoreceptors in the raphe nucleus. This is unlikely because the net effect of acute citalopram is an increase of extracellular serotonin levels, although the increase is not as great as that introduced by chronic citalopram or by a combination of citalopram and 5-HT autoreceptor antagonists (Moret and Briley, 1996).

We are also aware of the differences between acute and chronic treatments of SSRIs. In the context of depression, single-dose SSRIs enhanced positive biases (e.g. citalopram) and decreased threat-relevant biases (e.g. mirtazapine) in emotional perception and memory (Harmer *et al.*, 2003; Browning *et al.*, 2007; Arnone *et al.*, 2009; Rawlings *et al.*, 2010). However, clinical effects of SSRIs are usually observed after a couple of weeks of chronic treatment. The delay in the clinical effects of SSRIs may be because of the relearning of emotional association, a process which takes time and exposure to a more positive emotional environment (Harmer and Cowen, 2013). In fear conditioning tasks, acute and chronic SSRI administrations even produced opposite effects (Burghardt *et al.*, 2004).

In the alternative context of impulse control, acute citalopram treatments benefit NoGo type (for a review, see Eagle *et al.*, 2008) and other forms of response inhibition in animal models (Eagle *et al.*, 2009; Miyazaki *et al.*, 2011; Baarendse and Vanderschuren, 2012; Humpston *et al.*, 2013). In healthy adults, the acute effect of citalopram was observed on frontal activations but not always in behaviour (Del-Ben *et al.*, 2005; Chamberlain *et al.*, 2006; Macoveanu *et al.*, 2013) while recognizing the complexity of serotonergic systems in impulsive action (Kirby *et al.*, 2011). On tasks that require inhibition of response sets, such as reversal learning task, the acute and chronic effects of citalopram are comparable (Danet *et al.*, 2012). However, the acute and chronic treatments may have different mechanisms, either pharmacologically or in terms of the networks and neural targets they engage. For example, single-dose citalopram may reduce impulsive actions by directly increasing extracellular serotonin levels in the prefrontal cortex, as we suggest is likely to occur in this study. But we cannot rule out stimulation of frontal 5-HT_2A_ receptors as occurs in chronic citalopram treatment (Furr *et al.*, 2012). 

It has been proposed that the seemingly separate roles of serotonin in depression and inhibition are indeed related (Cools *et al.*, 2011). For example, recent studies suggested that the link between negative mood and serotonin decrease is indirect and likely mediated by damaged inhibition and/or through associative learning (Dayan and Huys, 2008; Robinson and Sahakian, 2008). Other studies showed that serotonin mediates behavioural inhibition and impulsivity in an affective context (Cools *et al.*, 2005; Crockett *et al.*, 2009).

Serotonin has also been linked with semantic memory (Mendelsohn *et al.*, 2009). We examined the effect of citalopram on category fluency (as measured by the number of words produced in a category), which was impaired in Parkinson’s disease (Table 1). A repeated-measures ANOVA revealed an interaction between drug and disease severity [*F*(1,14) = 7.56, *P < *0.05], indicating that citalopram improved category fluency in patients with more advanced disease. Citalopram may facilitate the inhibition of simultaneously active conceptual representations during lexical selection, which is mediated by the inferior frontal cortex (Badre and Wagner, 2007), rather than the simple articulatory process. In support of this, letter fluency, which requires articulatory process but not selection among concepts, was not improved by citalopram. However, a single framework that integrates the roles of neurotransmitters in cognitive control and language processing is beyond the scope of this study. Further studies are needed to test to what extent neurocognitive models of attentional control in perception and action can explain inhibition and shifting in comprehension and production of word, sentence and discourse.

This study has several methodological limitations. First, we used functional MRI blood oxygen level-dependent signals, which could in principle be influenced by citalopram. However, this is unlikely because frontal regions retain normal blood flow on a NoGo task after an acute treatment of citalopram (Macoveanu *et al.*, 2013). Second, we focused on the right inferior frontal gyrus because this region is consistently associated with motor inhibition in many different versions of NoGo and Stop-Signal tasks (Rubia *et al.*, 2001). The right inferior frontal gyrus may also provide a cortical ‘readout’ of subcortical effects (Agnoli and Carli, 2012): it would be interesting to examine the effects of citalopram on the striatum but the striatum had relatively poor signal-to-noise ratio compared to cortical regions using the quiet-EPI sequence. A role of the striatum in mediating the effects of citalopram, in conjunction with prefrontal cortex, is, however, suggested by diffusion MRI that the preserved structural connectivity between frontal and striatal regions was a significant predictor of the effectiveness of citalopram treatment. Third, Parkinson’s disease may also affect other processes of cognitive control on NoGo trials, such as action selection, in addition to inhibition (Hughes *et al.*, 2010). Fourth, the heterogeneity of Parkinson’s disease may reduce the power of group-level analysis. We therefore used a powerful within-subject design and emphasized individual differences, together with strong control of the type I error. However, we cannot rule out a type II error, especially for the lack of effects of disease and citalopram on NoGo trials, as the activity pattern of NoGo was less extensive and less intensive even in healthy controls. Still the positive results of this study are large by comparison with many functional MRI studies in Parkinson’s disease. Fifth, our patients were in the mid-stage of Parkinson’s disease, but all were taking dopaminergic medication (81% were taking dopamine receptor agonists). To identify the effect of dopamine agonists, we conducted a *post hoc* ANOVA with target drug (citalopram versus placebo) and dopamine agonist (taking versus not taking). There was no interaction for either NoGo (*P = *0.52) or Stop-Signal trials (*P = *0.27), suggesting that the effect of citalopram is not strongly influenced by concurrent dopamine agonists. We also cannot comment on the application of citalopram for drug-naïve patients immediately after diagnosis, except to note that the positive correlation between citalopram-induced reduction of NoGo error and levodopa equivalent dose suggests that those patients may also benefit from citalopram, at least on action restraint. Finally, we did not include patients with impulse control disorders although impulse control disorder was not an exclusion criterion. This reflects perhaps the low prevalence and high awareness of impulse control disorders in a specialist service, combined with possible patient recruitment biases regarding patients with severe impulse control disorders.

In conclusion, we have shown that the selective serotonin reuptake inhibitor citalopram can improve behavioural impulsivity in terms of action restraint and cancellation, but only in a subset of patients with Parkinson’s disease (patients with relatively more severe disease). The behavioural benefits of citalopram relate to enhanced activations in the inferior frontal cortex. These findings contribute to the broader understanding of the role of serotonergic systems in regulating cognitive and behavioural control, and indicate the need for patient stratification in clinical trials of serotonergic treatments in Parkinson’s disease.

## Supplementary Material

Supplementary Data

## Abbreviations

SSRI: selective serotonin reuptake inhibitor
SSRT: Stop-Signal Reaction Time
UPDRS: Unified Parkinson’s Disease Rating Scale
