# Supplementary material for: Selective serotonin reuptake inhibition modulates response inhibition in Parkinson’s disease
Source: Brain. 2014 Feb 27;137(4):1145–55. doi: 10.1093/brain/awu032 (PMC3959561; doi:10.1093/brain/awu032)
Supplement: Supplementary Data [file supp_137_4_1145__index.html]

Selective serotonin reuptake inhibition modulates response inhibition in Parkinson’s disease — Supplementary Data 

# Selective serotonin reuptake inhibition modulates response inhibition in Parkinson’s disease

## Supplementary Data

files

**Files in this Data Supplement:**

- Supplementary Data - docx file
