# Supplementary material for: Selective serotonin reuptake inhibition modulates response inhibition in Parkinson’s disease
Source: Brain. 2014 Feb 27;137(4):1145–55. doi: 10.1093/brain/awu032 (PMC3959561; doi:10.1093/brain/awu032)
Supplement: Supplementary Data [file supp_awu032_brain-2013-01563-File006.docx]

**Supplemental Materials**

Neuropsychological tests

The simple and choice reaction time tasks assess general alertness and motor speed.

In the CANTAB simple reaction time task, subjects responded to the appearance of a white square on the screen by pressing the button on the press pad. Duration ~6 minutes.

In the CANTAB choice reaction time task, an arrow-shaped stimulus was displayed on either the left or the right side of the screen, pointing left of right. Subjects were asked to press the left hand button on the press pad if the stimulus was displayed on the left hand side of the screen, and the right hand button on the press pad if the stimulus was displayed on the right hand side of the screen. Duration ~7 minutes.

Effects of Citalopram in SMA

The Stop-Signal and NoGo tasks were also associated with activation of the supplementary motor area (SMA) in our study and previous reports. We therefore investigated *post hoc* the effects of Citalopram on the SMA.

The SMA ROI was defined as the intersection of the AAL-based anatomical SMA and the SS>Go activation in controls (one-sample t-test, voxel-level p<0.001 uncorrected, cluster-level p<0.05 FWE-corrected). The ROI is therefore independent the patient data with which we sought an effect of drug. Parameter estimates (beta values in SPM) of SS>Go and NoGo>Go in PD were entered into repeated-measures ANOVAs with drug as a within-subject factor and UPDRS-motor, age, LED and plasma concentration as covariates (similar to the RIFG ROI analysis in the main text). There was no significant effect of Citalopram in the SMA.

Despite the activation of pre-supplementary motor area (pre-SMA) in some previous fMRI studies of response inhibition, we did not find the pre-SMA activation in our healthy controls (at exploratory threshold, voxel-level p<0.001 uncorrected, cluster-level p<0.05 uncorrected).
